# Novel High-Energy-Efficiency AlGaN/GaN HEMT with High Gate and Multi-Recessed Buffer

**DOI:** 10.3390/mi10070444

**Published:** 2019-07-02

**Authors:** Shunwei Zhu, Hujun Jia, Tao Li, Yibo Tong, Yuan Liang, Xingyu Wang, Tonghui Zeng, Yintang Yang

**Affiliations:** School of Microelectronics, Xidian University, Xi’an 710071, China

**Keywords:** GaN, HEMT, high gate, multi-recessed buffer, power density, power-added efficiency

## Abstract

A novel AlGaN/GaN high-electron-mobility transistor (HEMT) with a high gate and a multi-recessed buffer (HGMRB) for high-energy-efficiency applications is proposed, and the mechanism of the device is investigated using technology computer aided design (TCAD) Sentaurus and advanced design system (ADS) simulations. The gate of the new structure is 5 nm higher than the barrier layer, and the buffer layer has two recessed regions in the buffer layer. The TCAD simulation results show that the maximum drain saturation current and transconductance of the HGMRB HEMT decreases slightly, but the breakdown voltage increases by 16.7%, while the gate-to-source capacitance decreases by 17%. The new structure has a better gain than the conventional HEMT. In radio frequency (RF) simulation, the results show that the HGMRB HEMT has 90.8%, 89.3%, and 84.4% power-added efficiency (PAE) at 600 MHz, 1.2 GHz, and 2.4 GHz, respectively, which ensures a large output power density. Overall, the results show that the HGMRB HEMT is a better prospect for high energy efficiency than the conventional HEMT.

## 1. Introduction

Wide-bandgap semiconductor materials exhibit many attractive properties far beyond the capabilities of silicon, such as high critical breakdown electric field strength, carrier drift velocity, high thermal conductivity, and large carrier mobility. Therefore, power electronic devices based on wide-bandgap semiconductor materials such as diamond, silicon carbide (SiC), and gallium nitride (GaN) will have higher resistance to high voltages, and lower on-resistance and radiation resistance than silicon devices [1,2,3,4]. Recently, GaN devices became a research hotspot of high-frequency and high-power devices and systems with its large forbidden band width, high electron saturation speed, high breakdown voltage, and anti-irradiation [5,6,7,8,9]. The wide-bandgap semiconductor device GaN high-electron-mobility transistor (GaN HEMT) has the advantages of high frequency, high power density, high withstand voltage, and high efficiency; thus, it is used in civil communication, Internet of things, petroleum exploration, aerospace, and so on [10,11]. However, traditional GaN HEMTs are unable to meet the current demand. At present, most research on GaN HEMTs is based on peripheral circuits to regulate and compensate transistors to achieve better output characteristics [12]. However, such designs often lead to shortcomings such as poor transistor withstand voltage, large parasitic capacitance, and a narrow transconductance saturation region, which have a great influence on important performance parameters such as output power and power-added efficiency of the device.

Based on the conventional GaN HEMT, a novel AlGaN/GaN HEMT with a high gate and a multi-recessed buffer (HGMRB HEMT) is proposed in this paper for high-energy-efficiency applications. Compared with its conventional counterpart, the high gate and multi-recessed buffer structure changes the electric field distribution, the gate source capacitance, and the transconductance parameters of the HEMT.

## 2. Device Structure and Description

**Figure 1:**
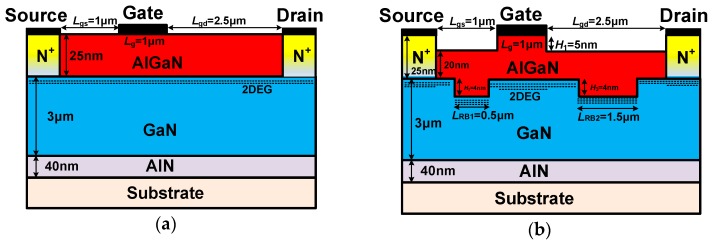
Schematic cross sections of the (**a**) conventional high-electron-mobility transistor (HEMT), and (**b**) proposed HEMT with a high gate and a multi-recessed buffer (HGMRB).

Figure 1 shows the device structure of the conventional HEMT (a) and the HGMRB HEMT (b). The source and drain of both devices are N^+^ heavily doped with a doping concentration of 1 × 10^20^ cm^−3^ and have the same 40 nm AlN nuclear layer and SiC substrate layer with a lateral width of 6.5 μm. The self-heating effect is one of the main reasons for restricting GaN devices. It not only affects the output power, but also affects the reliability of the device. Many methods for reducing the self-heating effect were reported, such as changing the substrate material [13,14]. In this paper, SiC is used as the substrate, which greatly reduces the self-heating effect. Compared with the conventional HEMT, the barrier region of the proposed HGMRB HEMT is 5 nm lower than the source, drain, and gate electrodes, forming a high gate. The barrier region between the source/drain and the high gate has a height of 20 nm. The buffer layer height of both devices is 3 μm. The buffer layer of the HGMRB HEMT forms two left and right recessed regions. The widths of the recessed regions are 0.5 μm and 1.5 μm, respectively, and the depth is 4 nm.

In the proposed new structure, the distribution of the electric field can reduce the electric field peak at the edge of the gate electrode and reduce the electron injection effect near the gate, and the current collapse effect can be alleviated. Therefore, the effect of surface state on device performance is improved. The negative impact of damage caused by etching on device performance is slight under the existing process equipment conditions. In metal-oxide-semiconductor (MOS) devices, carriers flow through the channel region. If the channel region is very narrow, quantum effects need to be considered; however, in the proposed HEMT, electrons flow mainly under the recessed region, rather than in the recessed region. The recessed regions in this paper are mainly used to change the capacitors (*C*_gs_, *C*_gd_, and so on) and the two-dimensional electron gas distribution of the device. Therefore, there is no need to consider the quantum effect.

## 3. Results and Discussion

The novel AlGaN/GaN HEMT with a high gate and a multi-recessed buffer was simulated using the TCAD Sentaurus software, and the physical model and key parameters used in the simulation are shown in Table 1. By measuring the traditional HEMT, the values of carrier mobility *μ*, *N*_c_, and *N*_v_ were obtained, and other material parameters were default values. The mobility defines the carrier mobility models, which include electron mobility degradation due to high doping. The thermodynamic model extends the drift–diffusion approach to account for electrothermal effects under the assumption that charge carriers are in thermal equilibrium with the lattice. The effective intrinsic density triggers the bandgap-narrowing effect in highly doped regions using the specified model OldSlotboom. Shockley–Read–Hall (SRH) recombination is activated within the recombination. The Fermi activates the carrier Fermi statistics. Incomplete ionization must be considered when impurity levels are relatively deep compared to the thermal energy (*kT*). The solution model is coupled {Poisson electron hole}, and the initial temperature is set to 300 K by default in simulations [15]. In the ADS software, the EE_HEMT model [16] was used. The criterion of breakdown was BreakCriteria {Current (Contact = “Drain” Absval = 1 × 10^−4^)}. In order to get a more accurate calculation result, the number of iterations was set to 50, and the error reference of the electron was set to 1 × 10^−3^. The parameters in the EE_HEMT model were obtained from the TCAD simulations, known literature, and technical manuals, the gate was the input terminal, and the drain was the output terminal, in the different frequency bands of 600 MHz, 1.2 GHz, and 2.4 GHz. The simulation results obtained using Synopsys TCAD Sentaurus and ADS software show that the new structure has better RF characteristics and greater power-added efficiency (PAE).

### 3.1. Direct Current (DC) Characteristics

**Figure 2:**
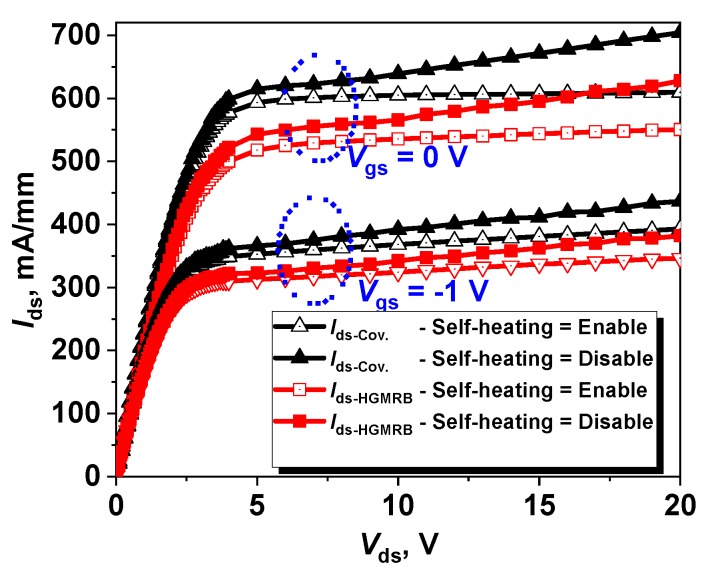
Output characteristics under different values of gate bias for the conventional HEMT and HGMRB HEMT including self-heating.

It can be seen from Figure 2 that, under a large drain bias, a large current will cause the crystal lattice to heat up, forming a self-heating effect. When *V*_gs_ = 0 V, the effect of self-heating on the output characteristics is more obvious; thus, the self-heating effect must be considered. The drain saturation current of the HGMRB HEMT is slightly smaller than that of the conventional HEMT. At *V*_gs_ = 0 V and *V*_ds_ = 20 V, the maximum drain saturation currents of the HGMRB HEMT and conventional HEMT were 550.26 mA/mm and 609.32 mA/mm, respectively, which were reduced by 59 mA/mm, whereby the saturated drain current of the new structure was 9.68% lower than the conventional structure. Similarly, when *V*_gs_ = 0 V and *V*_ds_ = −1 V, the saturated drain current of the new structure was 11.73% lower than the conventional structure Since the HGMRB structure is used, there are two recessed regions in the channel region, and the discontinuous channel region hinders the channel current; the deeper the recess depth of the buffer region is, the smaller the channel current will be. At the same time, the Two-dimensional electron gas (2DEG) concentration of the channel region is proportional to the thickness of the barrier layer. The barrier layer of the HGMRB structure will be smaller than the conventional HEMT, resulting in a decrease in channel current. Combining the two points above, the maximum drain saturation current of HGMRB was slightly smaller than that of the conventional HEMT. In order to keep the channel current from dropping significantly, two recesses were formed in the buffer region, and the depth of the recessed region was not particularly large.

**Figure 3:**
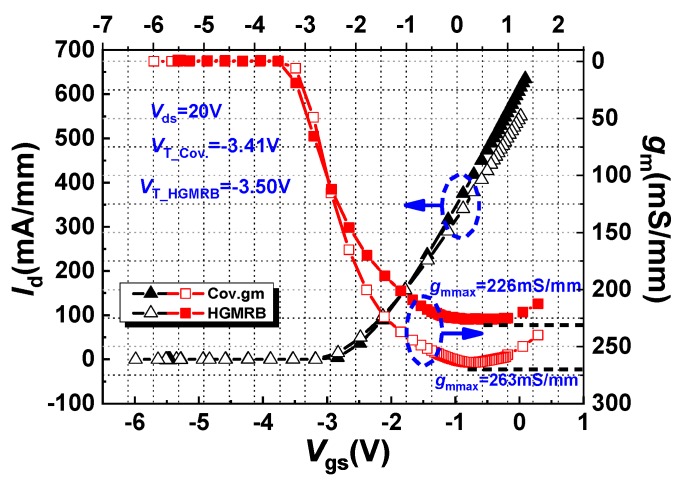
Transconductance and transfer curve with gate voltage at *V*_ds_ = 20 V.

Figure 3 shows the transfer characteristics and transconductance curves of the conventional HEMT and the HGMRB HEMT at *V*_ds_ = 20 V. It can be seen from Figure 3 that the threshold voltages *V*_t_ of the conventional HEMT and the HGMRB HEMT were −3.41 V and −3.50 V, respectively. As the gate voltage approached 0 V, the drain currents of both HEMTs gradually increased, and the drain current value of HGMRB was smaller than that of the conventional HEMT. Since the magnitude of the threshold voltage is related to the thickness of the barrier layer under the gate, when the thickness of the barrier layer is the same, the depletion region formed under the gate is almost uniform; thus, the turn-on voltages of the two devices are almost identical.

According to the definition of transconductance *g*_m_, transconductance refers to the ratio between the change value of the current at the output end and the change value of the input terminal voltage. The first-order derivation of the transfer curve is shown in Figure 3. From the figure, the voltage control range of the HGMRB HEMT was slightly stronger than that of the conventional HEMT, but the maximum transconductance *g*_mmax_ was 37 mS/mm smaller than the conventional HEMT. Due to the existence of a recessed area on the surface of the buffer layer where the channel region was located, the channel region was not flat, and the maximum saturated drain current was reduced; however, the existence of the recess could increase the thickness of the barrier layer above the recess, resulting in an increase in 2DEG, and this kept the maximum drain saturation current from being too low.

**Figure 4:**
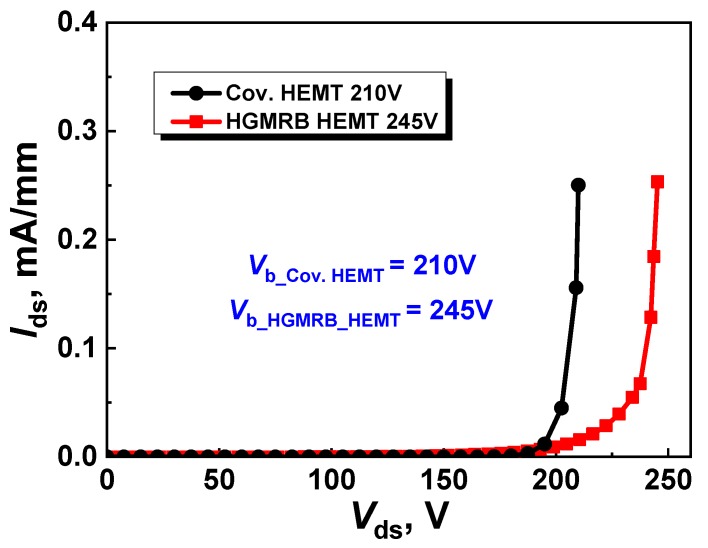
Breakdown characteristics of the two devices at *V*_gs_ = *V*_t_.

**Figure 5:**
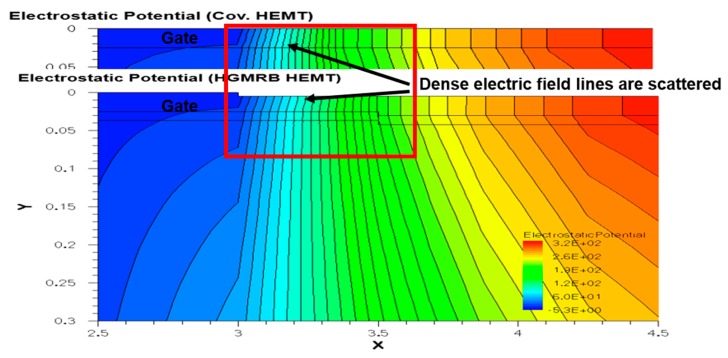
Electrostatic potential distribution of the conventional HEMT and proposed HGMRB HEMT.

Figure 4 shows the breakdown characteristics of the device at *V*_gs_ = *V*_t_, where the break criterion is that the absolute value of the gate is 1 × 10^−7^ A. The results show that the breakdown voltages (*V*_b_) of the conventional HEMT and HGMRB HEMT were 210 V and 245 V, respectively, with the breakdown voltage increasing by 16.7%. When the high drain voltage was applied, a high electric field was formed at the edge of the gate, such that, when the drain voltage reached a certain value, breakdown occurred at the position of the gate of the GaN HEMT near the drain side. Figure 5 shows the electrostatic potential distribution of the two devices, where it can be seen that the equipotential line distribution on the right side of the gate of the conventional HEMT (*x* > 3) changed to dense firstly and then to sparse, while, in the HGMRB HEMT, the equipotential lines on the right side of the gate (*x* > 3) were more evenly distributed and were not particularly dense, which effectively slowed down the electric field concentration near the gate, enabling the HGMRB HEMT to withstand larger drain voltages and improve the breakdown voltage of the device.

### 3.2. RF Characteristics

**Figure 6:**
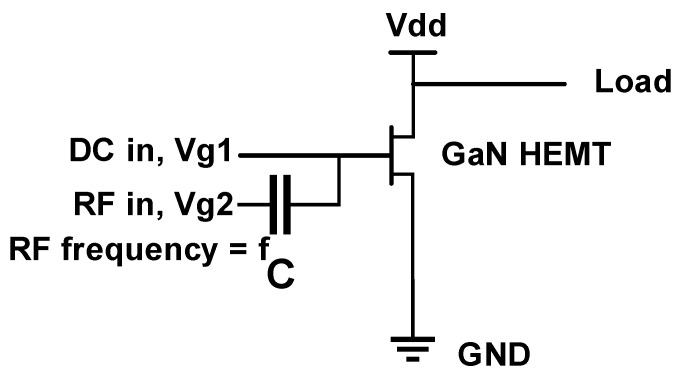
One-tone load-pull schematic for measurements.

**Figure 7:**
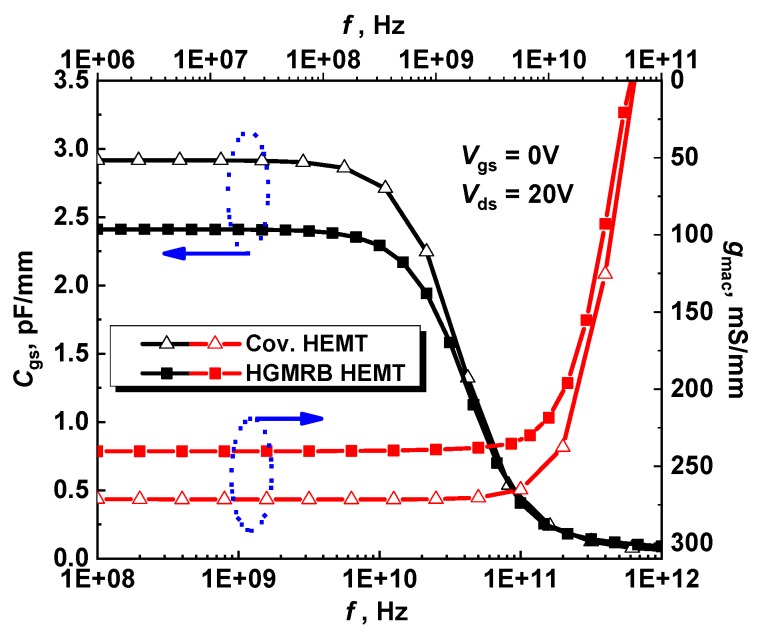
Gate-source capacitance (*C*_gs_) and transconductance (*g*_m_) versus frequency of the two devices at *V*_gs_ = 0V, *V*_ds_ = 20V.

The device was biased as shown in Figure 6. The gate was the input terminal in the different frequency bands, and the drain was the output terminal. Figure 7 shows the curve of the gate source capacitance (*C*_gs_) and alternating current (AC) transconductance versus frequency for the two devices with *V*_gs_ = 0 V and *V*_ds_ = 20 V. Under this bias condition, the DC operating point of the device was better, which is beneficial to obtain more accurate parameters. The simulation results show that when the frequency was 1 GHz, the *C*_gs_ values of the conventional HEMT and HGMRB HEMT were 2794.49 pF/mm and 2410.57 pF/mm, respectively, and the *C*_gs_ value of the new structure was about 506 pF/mm lower than the conventional structure. Due to the existence of the high gate, when *V*_gs_ = 0 V, the depletion region could only diffuse vertically downward [17], while the depletion region under the conventional structure gate diffused to both the source and the drain, and the capacitance area increased [18]. The simulation results show that the depth of the depletion region below the gate of the new structure was deeper than that of the conventional structure. According to the definition of the parallel plate capacitor [19], the gate capacitance of the new structure can be lower than that of the conventional structure.

(1) $$C=\frac{\varepsilon_{0}S}{4\pi kd}$$

From the AC transconductance curves of the two structures, it can be seen from the figure that the AC transconductance value of the conventional HEMT device was 31.00 mS/mm higher than that of the HGMRB HEMT at *V*_gs_ = 0 V and *V*_ds_ = 20 V, and the AC transconductance peak of the HGMRB HEMT was 240.31 mS/mm. The transconductance peak value under DC conditions was 10.30 mS/mm, and the AC peak transconductance of the conventional HEMT device increased by 14.00 mS/mm.

In AC conditions, the RF signal loaded on the gate periodically changed with frequency, such that the channel output current also changed periodically. When the frequency signal change period exceeded the time constant, the channel current could be changed in the future in the same signal period, resulting in a decrease in current and a decrease in the AC transconductance value under high-frequency conditions.

**Figure 8:**
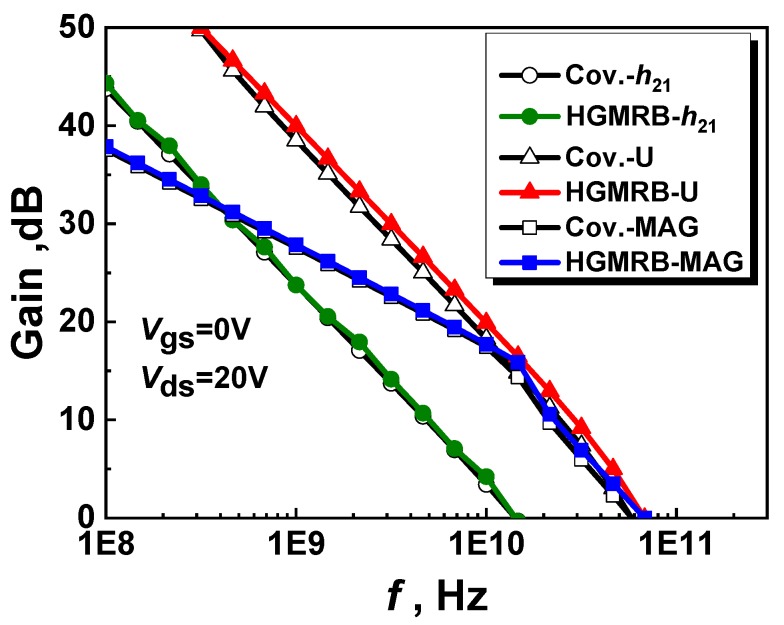
Small-signal high-frequency characteristic curve of two devices at *V*_gs_ = 0 V, *V*_ds_ = 20 V.

In order to obtain the cutoff frequency and maximum oscillation frequency of the HGMRB HEMT device, a two-port network was used for small-signal S-parameter simulation, in which *V*_gs_ = 0 V and *V*_ds_ = 20 V. Figure 8 shows the simulation results of the small-signal high-frequency characteristic curves of the two structures, where *h*_21_ is the small signal current gain of the device, maximum available gain (MAG) is the maximum gain of the device, and U is the unilateral power gain of the device. When *h*_21_ dropped to 0 dB, the cutoff frequency of the HGMRB HEMT and the conventional HEMT device was almost the same, and the cutoff frequency *f*_t_ was about 14.2 GHz. The cutoff frequency *f*_t_ is inversely proportional to the gate source capacitance *C*_gs_ and is proportional to the transconductance *g*_m_. Since the transconductance and the gate source capacitance of the HGMRB HEMT were simultaneously reduced, the drop in the transconductance peak of the device and the decrease in the capacitance of the gate source were offset by the effect of *f*_t_; thus, the cutoff frequency of the new structure hardly changed. When the unilateral gain U and the maximum achievable gain MAG dropped to 0 dB, the maximum oscillation frequencies *f*_max_ of the HGMRB HEMT and the conventional HEMT were about 66 GHz and 57 GHz, respectively, whereby the new structure was 15.78% higher than the traditional structure. It can be seen from Equation (3) that the HGMRB HEMT device itself had a smaller gate resistance value without changing *f*_t_, thus increasing *f*_max_.

(2) $$f_{t}\approx \frac{g_{m}}{2\pi C_{\mathrm{gs}}}$$

(3) $$f_{\max}=\frac{f_{t}}{2}\cdot \sqrt{\frac{R_{\mathrm{ds}}}{R_{g}}}$$

The device structure parameters described in Table 2 were obtained by simulation verification of Section 3.1 and Section 3.2, and these parameters were brought into the EE_HEMT model of the ADS software, and the energy efficiency verification was performed at different frequencies. The DC offset was *V*_gs_ = −4 V and *V*_ds_ = 20 V.

### 3.3. Verification of High Energy Efficiency

**Figure 9:**
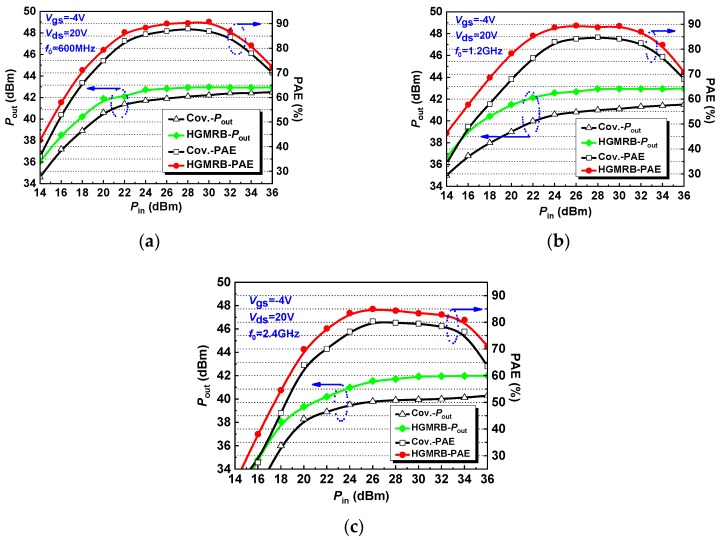
Large-signal performance of the two devices at different frequencies: (**a**) 600 MHz, (**b**) 1.2 GHz, and (**c**) 2.4 GHz.

Figure 9a shows the output power (*P*_out_) and power-added efficiency (PAE) as a function of input power (*P*_in_) for the HGMRB HEMT and conventional HEMT under *V*_gs_ = −4 V and *V*_ds_ = 20 V bias conditions at 600 MHz operating frequency. The results show that the power-added efficiency (PAE) of the HGMRB HEMT was always greater than the conventional HEMT. When the input power was 32 dBm, the output power of the HGMRB HEMT reached 42.92 dBm, the output power density was 9.8 W/mm, the power gain was 10.9 dB, and the power-added efficiency reached the maximum value of 90.8%, which was higher than the maximum additional efficiency of the conventional HEMT. When the operating frequency was increased to 1.2 GHz, the *P*_out_ and the PAE as a function of the input power *P*_in_ are shown in Figure 9b. When the input power was 32 dBm, the PAE of the HGMRB HEMT reached 87.0%, the *P*_out_ reached 42.93 dBm, and the power gain was 10.9 dB. At 1.2 GHz, the power output capability and power-added efficiency of the HGMRB HEMT were like those at 600 MHz, but the HGMRB HEMT had greater efficiency. Figure 9c shows the *P*_out_ and the PAE as a function of *P*_in_ at 2.4 GHz, *V*_gs_ = −4 V, and *V*_ds_ = 20 V. Due to the smaller gate source capacitance of the HGMRB HEMT, its advantages in saturated output power began to increase as the operating frequency continued to increase. When the input power reached 26 dBm, the PAE of the device was maximum, about 85.0%, and the *P*_out_ was 41.55 dBm. When the *P*_out_ was saturated, the output power was 41.95 dBm and the saturated output power density was 7.7 W/mm. The output power of the HGMRB HEMT was always greater than that of the conventional HEMT. 

Through the above analysis of the HGMRB HEMT and conventional HEMT, the HGMRB output power and PAE were always greater than conventional HEMT at the same input power. Because the HGMRB HEMT had the advantage of smaller gate source capacitance, when the frequency increased, the device’s advantages in power gain and PAE began to fully be reflected.

Table 3 shows the performance of the various parameters of the device at different frequencies in detail. Through the above analysis, HGMRB HEMT and conventional HEMT, HGMRB output power and PAE are always greater than conventional HEMT at the same input power, and because HGMRB HEMT has smaller gate-source capacitance advantages, along with frequency increased, the device’s advantages in power gain and PAE began to fully be reflected.

### 3.4. Key Process Steps for HGMRB HEMT

**Figure 10:**
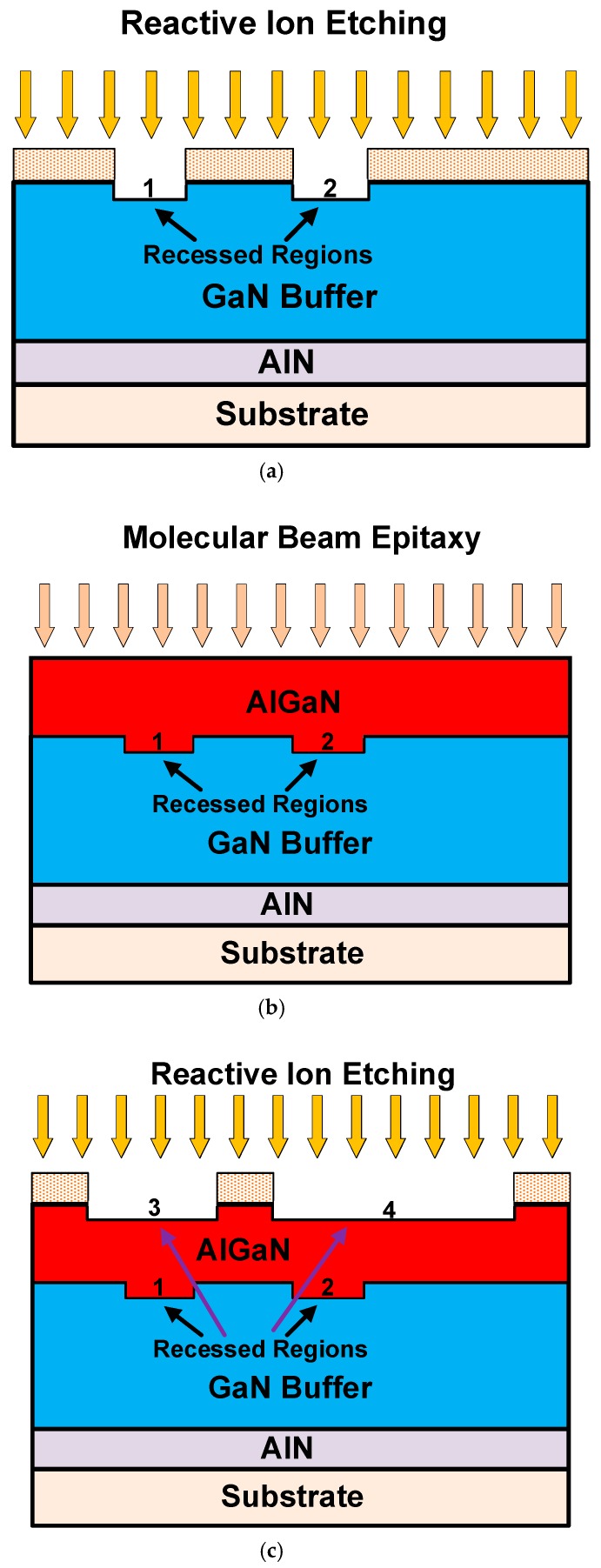

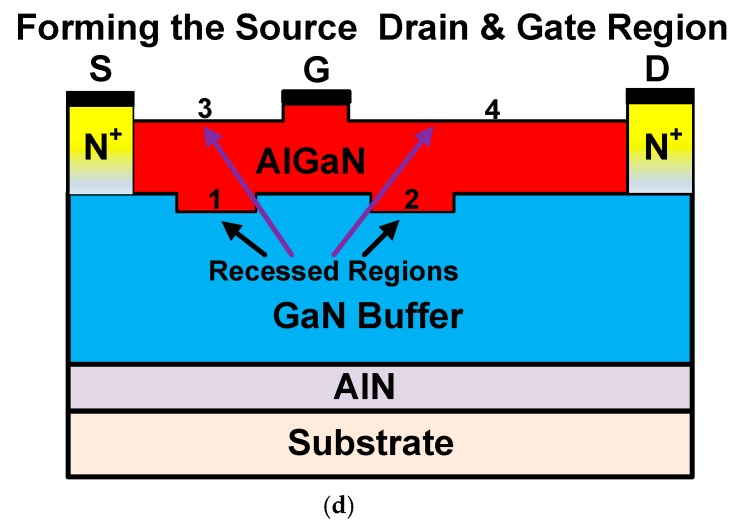
Key processes to fabricate the HGMRB HEMT. (**a**) Reactive ion etching is used to form the two recessed regions 1 and 2. (**b**)The AlGaN barrier layer is grown by molecular beam epitaxy. (**c**) Reactive ion etching is used to form the two recessed regions 3 and 4, and a high gate is obtained. (**d**) Source, drain, gate, and ohmic contact processes are formed.

A feasible key fabrication process is shown in Figure 10. Differing from the conventional HEMT process, a high gate and a multi-recessed buffer should be grown in the process below. Firstly, in Figure 10a, reactive ion etching (RIE) was used in the upper surface of the GaN layer of the device, and two recessed regions 1 and 2 were etched. Secondly, in Figure 10b, the AlGaN barrier layer was grown by molecular beam epitaxy (MBE). During the film growth process, the Al content, the impurity dose, and the overall thickness of the barrier layer were controlled in the barrier layer. Thirdly, in Figure 10c, reactive ion etching (RIE) was used. Photolithography was performed on both sides of the upper surface of the AlGaN layer of the device, and two recessed regions 3 and 4 were etched to form a high gate. Then, in Figure 10d, the source, drain, gate, and ohmic contact processes were formed the same as those of the conventional GaN HEMT, where the negative impact of damage caused by etching on device performance was slight under the existing process equipment conditions.

## 4. Conclusions

In this paper, a novel AlGaN/GaN HEMT with a high gate and a multi-recessed buffer for high-energy-efficiency applications was proposed and investigated using TCAD Sentaurus and ADS simulations. Although the maximum drain saturation current and transconductance slightly decreased, the breakdown voltage increased by 16.7%, the gate source capacitance dropped by 17% and the new structure had better gain and higher energy efficiency than the conventional HEMT. The output power density and PAE of the HGMRB HEMT were greater than those of the conventional HEMT in different frequency bands. The results show that the HGMRB HEMT is a better prospect for high energy efficiency than the conventional HEMT.

## Figures and Tables

**Table 1:** Key parameters used in simulation.

Physical Model	Descriptions	Key Parameters
Mobility	Carrier mobility	*μ* = 1.300e + 03 cm^2^/(Vs)
Thermodynamic	Lattice heat capacity	*c*_v_ = 3.0 J/(K·cm^3^)
	Lattice thermal conductivity	*κ* = 1.3 W/(K·cm)
Effective intrinsic density	*n*_i_(*T*) = *N*_C_(*T*) × *N*_V_(*T*) exp (*E*_g_(*T*)/(2*kT*)) *n*_i,eff_ = *n*_i_ × exp × (*E*_bgn_/(2*kT*))	*N*_C_ = *T*^3/2^ × 4.5 × 10^14^ *N*_V_ = *T*^3/2^ × 8.9 × 10^15^
Shockley–Read–Hall (SRH) recombination	*τ* = *τ*_min_ + (*τ*_max_ − *τ*_min_)/(1 + (*N*/*N*_ref_)	*τ*_min_ = 0.0000 s *τ*_max_ =1.0000 × 10^−9^ s *N*_ref_ = 1.0000 × 10^16^ cm^−3^
Fermi	*E*_g_ = *E*_g0_ + *α* × *T*_par_^2^/(*β* + *T*_par_) – *α* × *T*^2^/(*β* + *T*)	*E*_g0_ = 3.47 eV *α* = 7.40 × 10^−4^ eV/K *β* = 6.00 × 10^2^ K *T*_par_ = 0.0000 K

**Table 2:** Parameters of the conventional high-electron-mobility transistor (HEMT), and proposed HEMT with a high gate and a multi-recessed buffer (HGMRB).

Parameters	Conventional HEMT	HGMRB HEMT
*I*_dsat_ (mA/mm)	609.32	550.26
*V*_b_ (V)	210.00	314.00
*g*_m_ (mS/mm)	263.00	226.00
*V*_t_ (V)	−3.41	−3.50
*C*_gs_ (fF/mm)	2916.49	2410.57

**Table 3:** Comparison of output characteristics between the two structures at different frequencies. PAE—power-added efficiency.

Parameters	HGMRB HEMT			Conventional HEMT		
	600 MHz	1200 MHz	2400 MHz	600 MHz	1200 MHz	2400 MHz
Power density (W/mm)	9.8	9.8	7.7	8.7	6.7	5.0
Gain (dB)	10.9	10.9	9.9	10.4	9.3	8.0
PAE at saturation (%)	86.9	87.0	82.9	84.5	82.5	78.5
The maximum PAE (%)	90.8	89.3	84.4	88	84.7	80.3
